# COVID-19 Diagnosis in Computerized Tomography (CT) and X-ray Scans Using Capsule Neural Network

**DOI:** 10.3390/diagnostics13081484

**Published:** 2023-04-20

**Authors:** Andronicus A. Akinyelu, Bubacarr Bah

**Affiliations:** 1 Research Centre, African Institute for Mathematical Sciences (AIMS) South Africa, Cape Town 7945, South Africa; 2 Department of Computer Science and Informatics, University of the Free State, Phuthaditjhaba 9866, South Africa; 3 Department of Mathematical Sciences, Stellenbosch University, Cape Town 7945, South Africa

**Keywords:** COVID-19 diagnosis, medical imaging, capsule neural network, machine learning, CT scans

## Abstract

This study proposes a deep-learning-based solution (named CapsNetCovid) for COVID-19 diagnosis using a capsule neural network (CapsNet). CapsNets are robust for image rotations and affine transformations, which is advantageous when processing medical imaging datasets. This study presents a performance analysis of CapsNets on standard images and their augmented variants for binary and multi-class classification. CapsNetCovid was trained and evaluated on two COVID-19 datasets of CT images and X-ray images. It was also evaluated on eight augmented datasets. The results show that the proposed model achieved classification accuracy, precision, sensitivity, and F1-score of 99.929%, 99.887%, 100%, and 99.319%, respectively, for the CT images. It also achieved a classification accuracy, precision, sensitivity, and F1-score of 94.721%, 93.864%, 92.947%, and 93.386%, respectively, for the X-ray images. This study presents a comparative analysis between CapsNetCovid, CNN, DenseNet121, and ResNet50 in terms of their ability to correctly identify randomly transformed and rotated CT and X-ray images without the use of data augmentation techniques. The analysis shows that CapsNetCovid outperforms CNN, DenseNet121, and ResNet50 when trained and evaluated on CT and X-ray images without data augmentation. We hope that this research will aid in improving decision making and diagnostic accuracy of medical professionals when diagnosing COVID-19.

## 1. Introduction

Coronavirus disease 19 (COVID-19), one of the deadliest pandemics in the history of mankind, has swept through almost all the countries in the world [1]. Coronavirus has infected over 676 million people and killed over 6.88 million as of 17 March 2023, as indicated in the COVID-19 map of Johns Hopkins University. Unfortunately, the virus is still evolving, and new variants continue to emerge worldwide. Multiple nations, including Australia, Bangladesh, Denmark, India, Japan, and the United States, detected a novel immune-evasive COVID-19 strain (XBB) in August 2022, which is causing outbreaks in various nations. This shows that COVID-19 is still a threat, and there is a need for suitable techniques that can be used to tackle this pandemic.

Recently, computer-aided diagnosis technologies have become a fundamental part of routine clinical practice. These tools can be utilized to aid physicians in accurately diagnosing COVID-19 patients. Convolutional neural networks (CNNs) are one of the effective deep learning (DL) algorithms for building improved medical imaging systems. However, they are unable to handle input transformations effectively. In addition, CNNs must be trained on massive or augmented datasets to generate superior results. A capsule neural network (CapsNet) is a recent deep learning (DL) algorithm proposed by Hinton et al. [2]. CapsNets are resistant to image rotations and transformations [2], and they can produce excellent results when trained on small datasets [1,3]. 

This study proposes a CapsNet model for COVID-19 diagnosis using CT and X-ray images. This study also evaluates the robustness of CapsNets for image rotations and transformations. The main contributions of this study are as follows:

1. This study proposes an improved CapsNet technique for COVID-19 diagnosis (named CapsNetCovid). The proposed model was trained and evaluated on 14,000 CT images and 15,153 X-ray images. The results show that the proposed technique achieved good results for both CT and X-ray datasets. The full results are presented and discussed in Section 4.
2. As noted by the researchers that developed CapsNets [2], one of the key advantages of CapsNets over CNNs is its robustness to image rotations and affine transformations. To the best of the authors’ knowledge, no study has presented a performance analysis of CapsNet on different image rotations and transformations. This study presents a performance analysis of CapsNets on standard CT and X-ray images and their corresponding augmented variants. The analysis is presented for both binary classification and multi-class classification. The objective of the analysis is to evaluate the robustness of CapsNets to affine transformations.
3. To the best of the authors’ knowledge, no study has compared CapsNets and other CNN-based techniques in terms of their ability to recognize randomly transformed and rotated images. This study presents a comparative analysis between CapsNet, CNN, and two state-of-the-art CNN models, namely DenseNet121 and ResNet50. The comparative analysis is presented for both CT and X-ray images. The analysis compares the ability of CapsNet, CNN, DenseNet121, and ResNet50 to correctly identify randomly transformed and rotated CT and X-ray images without using data augmentation techniques.

This paper is organized as follows. A detailed review of related studies is presented in Section 2, and in Section 3, the methodology used in this study is presented. Furthermore, the dataset details and performance metrics used for evaluation are presented in Section 3. The results are presented and discussed in Section 4. The paper is finally concluded in Section 5.

## 2. Related Studies

Many DL-based COVID-19 diagnosis methods have been developed by different researchers, and most of them have produced promising results. AbouEl-Magd et al. [4] proposed a CNN-based COVID-19 diagnosis technique using VGG16 and a capsule neural network (CapsNet). The Synthetic Minority Oversampling Technique (SMOTE) [5] was used to generate new synthetic samples for their unbalanced dataset. Moreover, the Gaussian optimization technique was used to optimize the parameters for the CapsNet. Four different experiments were performed in the study. In the first two experiments, the CapsNet was evaluated on unbalanced datasets, as well as on balanced datasets based on class weights. In addition, the CapsNet was evaluated on a SMOTE-based balanced dataset. Finally, CapsNet was evaluated on a balanced dataset using the optimized parameters. The CapsNet model that was trained on the SMOTE-based balanced dataset produced the best classification accuracy and F1-scores of 96.73% and 97.08%, respectively.

Saif et al. [6] developed a modified CapsNet framework for COVID-19 diagnosis. The framework consists of three sets of convolution blocks concatenated in parallel. Each convolutional block consists of different filter sizes. Concatenating the convolutional blocks of different filter sizes helps to integrate discriminative coarse spatial features into the network. The proposed architecture handles images of large spatial resolution by using an extended number of capsule layers and routing numbers. The concatenated feature set is fed into the CapsNet. The CapsNet uses a concatenation technique in the capsule layers, in which the output of two higher-layer capsules is concatenated. The concatenated connections between the capsule layers helps to capture underlying complex features from specific regions in an image. Moreover, the concatenated connections strengthen the coupling coefficient and improves the learning ability of the capsule layers. It also increases the model’s ability to extract complex features from images with large spatial dimensions. The authors also used a pre-trained model to finetune the network’s performance. The proposed framework was tested on three different datasets, and it achieved classification accuracies of 96.0%, 96.8%, and 95.9% without finetuning. The technique also produced classification accuracies of 98.3%, 99.0%, and 98.9% for the three datasets with finetuning.

Toraman et al. [7] introduced a novel CapsNet architecture for COVID-19 diagnosis from X-ray images. The architecture is composed of five convolution layers, and each layer consist of 16, 32-, 64-, 128-, and 256-layer kernels. A large number of convolutional layers was added to provide effective feature maps to the primary layer of the CapsNet. The kernel size of the first three layers is 5 × 5, while the kernel size of the fourth layer is 9 × 9. The fifth layer is a primary capsule layer, consisting of 32 capsules, and each has a kernel size of 9 × 9. The proposed architecture was evaluated on a dataset consisting of 231 X-ray images of COVID-19 [8], 1050 X-ray images of no findings [9], and 1050 X-ray images of pneumonia [9]. The technique produced a classification accuracy of 84.22% and 97.24% for multi-class and binary classification, respectively. 

Tiwari et al. [10] designed a hybrid framework for COVID-19 diagnosis (called VGG-CapsNet). The framework consists of CapsNet and VGG16. The input images are fed into the VGG16 pre-trained network to extract feature maps. The extracted feature maps are then fed into the CapsNet for classification. The proposed technique was evaluated on a dataset containing 219, 1345, and 1341 radiography images of COVID-19, pneumonia, and normal conditions, respectively [11]. The proposed technique was evaluated for multi-class classification and binary classification. The proposed hybrid model was also compared with the standard CapsNet model (called CNN-CapsNet). The framework achieved 97% for binary classification and 92% for multi-class classification. The results also show that the proposed hybrid framework outperforms the standard CapsNet by 2% for binary classification and by 1% for multi-class classification.

Afshar et al. [12] proposed a CapsNet-based framework for COVID-19 diagnosis. The framework consists of four convolutional layers and three capsule layers. The first convolutional layer is followed by a batch normalization layer, while the second convolutional layer is followed by an average pooling layer. The features from the fourth convolutional layer are reshaped and fed into the CapsNet. The dataset used to evaluate the framework is imbalanced; the number of positive cases is lower than the number of negative cases. Therefore, the loss function of the network is modified, such that more weight is assigned to the positive samples. The weights are determined using a formula specified in [12]. The framework was evaluated by first pre-training it on a dataset containing 94,323 frontal view of chest X-ray images. The pre-trained network was then finetuned on a dataset containing 358 CXR COVID-19 images, 8,066 8,066 normal images, and 5538 non-COVID-19 images. The framework achieved an accuracy, sensitivity, specificity, and AUC of 95.7%, 90%, 95.8%, and 0.97, respectively.

Heidarian et al. [13] proposed a fully automated two-stage framework for COVID-19 diagnosis using CapsNet and CT images, called COVID-FACT. At the first stage, COVID-FACT uses U-Net architecture to detect infected slices from a 3D volumetric CT scan. The infected slices are classified in the second stage. Two variants of the framework were developed in the study. Whole CT images are used as inputs to the first variant, while the segmented lung region is used as an input to the second variant. COVID-FACT was trained on a dataset containing 171, 60, and 76 COVID-19, community-acquired pneumonia (CAP), and normal volumetric CT images, respectively. Experiments shows that the two variants produced the same classification accuracy of 90.82%. However, the variant that was trained on the segmented lung regions improved the sensitivity and AUC of the model by over 1.83% and 0.03, respectively. 

Quan et al. [14] designed a COVID-19 diagnosis method using DenseNet121 and CapsNet. They also introduced a dataset pre-processing technique that reduces the impact of dataset heterogeneity on the performance of a network. Data augmentation was also used to generate more datasets. The proposed framework uses a segmentation network, namely TernausNet [15], to segment or extract the lung contour from X-ray images. The segmented lung contours are then fed into DenseNet121 for feature extraction. The extracted features are fed into CapsNet for classification. The segmentation network was trained on the Montgomery County Chest X-ray Database [16], containing 80 and 50 normal and tuberculosis X-ray images, respectively. The classification network was trained on a dataset from three sources. The dataset contains 781, 2917, 2884, and 2850 COVID-19, normal, pneumonia (virus), and pneumonia (bacteria) X-ray images, respectively. The framework achieved a classification accuracy, sensitivity, and F1-score of 90.7%, 96%, and 90.9%, respectively.

Qi et al. [17] developed a fully automated pipeline for classifying COVID-19 from CAP using CT images. The pipeline consists of four modules. The first module uses LinkNet [18] to segment the lungs from CT images, and the second module uses CapsNet to select slices with lesions. The third module uses ResNet50 and CapsNet for slice-level prediction, and the fourth module uses DensNet121 and CapsNet for patient-level prediction. The pipeline was trained on a dataset containing 161 CT images with COVID-19 and 100 CT images with CAP. The CapsNet with ResNet50 achieved a classification accuracy and AUC of 92.5% and 0.933, respectively, for the slice-level prediction. The CapsNet with DenseNet121 achieved a better classification accuracy and AUC of 97.1% and 0.992, respectively, for slice-level prediction. The pipeline achieved an accuracy of 100% for patient-level prediction. 

Attallah [19] proposed a CNN-based technique for COVID-19 diagnosis called RADIC. RADIC is divided into four stages. In the first stage, four radiomics methods are used to analyze CT and X-ray images, including gray-level run-length matrix (GLRLM), gray-level covariance matrix (GLCM), discrete wavelet transform (DWT), and dual-tree complex wavelet transform (DTCWT). The output of the analysis was then converted to heatmap images. In the second stage, the heatmap images are used to train three CNN models, including MobileNet, DenseNet201, and Darknet53. After training, deep features were extracted from the batch normalization layers of the three models. Furthermore, the complexity of the extracted features was reduced using the fast Walsh–Hadamard transform (FWHT). The reduced features from the three CNN models were combined using discrete cosine transform. Finally, the combined features were used to train different classification models, including linear support vector machine (L-SVM), quadratic-SVM, linear discriminant analysis (LDA), and ensemble subspace discriminant (ESD). The technique was evaluated on a CT and X-ray dataset, and it produced 99.4% and 99% on the two datasets.

Mercaldo et al. [20] designed a DL technique for COVID-19 diagnosis using VGG16. They added one more fully connected layer to the VGG16 and trained the added layer on a dataset containing 18,000 CT images. The model achieved an accuracy of 95%. In another study, Shah et al. [21] designed a CNN-based technique for COVID-19 diagnosis. They evaluated the model on 738 CT images, and it produced a classification accuracy of 82.1%. They also compared the performance of the proposed model to DenseNet169, VGG16, ResNet50, InceptionV3, and VGG19. The comparison shows that VGG-19 outperformed the other techniques, achieving an accuracy of 94.52%.

Attallah and Samir [22] designed a DL-based pipeline for COVID-19 diagnosis using a multilevel discrete wavelet decomposition (DWT) and three ResNet models. DWT was used to analyze CT scans and generate heatmap images. The heatmap images were used to train three ResNet models. After training, spectral–temporal features were extracted from the three ResNet models, including ResNet50, ResNet101, and ResNet18. Furthermore, the same ResNet models were trained on the original CT images and some spatial features were extracted from the models after training. Furthermore, the spatial features were combined with the spectral–temporal features, and the combined feature dimension was reduced. Finally, the reduced features were used to train three SVM models. The technique was evaluated on two datasets, which achieved a classification accuracy of 99.33% and 99.7%.

Attallah [23] proposed a framework for COVID-19 diagnosis using texture-based radiomic images. The author trained three ResNet models (ResNet18, ResNet50, and ResNet101) on two types of texture-based radiomic images. The first set of images were generated by discrete wavelet transform, while the second set were generated by gray-level covariance matrix. After training, some texture-based radiomic features were extracted from the trained models and combined using discrete cosine transform. The fused features were used to train three SVM algorithms. The technique was evaluated on a dataset consisting of 2482 COVID-19 normal CT images, and it achieved a classification accuracy of 99.60%. Zhao et al. [24] designed a technique for COVID-19 diagnosis using a modified version of the ResNet model. In the modified model, the authors substituted group normalization for batch normalization and performed a weight standardization for all the convolutional layers. The model was evaluated on a dataset containing 194,922 images, and it achieved a classification accuracy of 99.2%.

Shankar and Perumal [25] proposed a novel technique for COVID-19 diagnosis. This technique is divided into three stages. In the first stage, Gaussian filtering is used for smoothening and noise removal from the images. Furthermore, the proposed fusion model is used to extract a different set of features from the processed images. It extracts handcrafted features using the local binary pattern model and DL features using the InceptionV3 model. Furthermore, the extracted features were fused and trained on the multilayer perceptron classifier. The technique was evaluated on an X-ray dataset consisting of 27 normal, 220 COVID-19, 11 SARS, and 15 Pneumocystis images, and it produced a classification accuracy of 94.08%. In another study, Marios et al. [26] presented an analysis of five DL algorithms for COVID-19 diagnosis using ResNet50, ResNet101, DenseNet121, DenseNet169, and InceptionV3. The models were trained on a dataset consisting 11,956 COVID-19 X-ray images, 10,701 normal images, and 11,263 pneumonia images. The results show that ResNet101 achieved the best classification accuracy of 96%. Attallah [27] proposed a CNN-based method for COVID-19 diagnosis using spectral–temporal images. This method is divided into three stages. In the first stage, multilevel discrete wavelet transform (DWT) is used to analyze CT images and extract spectral–temporal images. The extracted images were then used to train three ResNet models. After training, deep features were extracted and fused together. The dimension of the fused features was reduced and used to train SVM. The technique was evaluated on a dataset consisting of CT images, and it produced satisfactory accuracy. A summary of the literature review is presented in Table 1.

### Limitations of Existing COVID-19 Diagnosis Models

As shown in the summary and in a literature survey written in [28,29], COVID-19 diagnosis models have some shortcomings. Tracking people that are infected with COVID-19 is a challenging task. Moreover, identifying patients infected with COVID-19 beforehand is impossible because COVID-19 has an incubation period of 14 days. Furthermore, some of the datasets used for training lacks quality, as some of them are available in an unstructured format. In addition, some of the datasets are too clean, lacking representation of real-world datasets [28]. Moreover, the generalization performance of some of the proposed models is not good due to overfitting. Furthermore, most studies do not explore the use of unsupervised ML algorithms for COVID-19 diagnosis, such as principal component analysis (PCA) and cluster analysis [29]. Most studies also focus on DL algorithms, such as CNN, while few studies explored CapsNet. Furthermore, to the best of the authors’ knowledge, no study presented a performance analysis of CapsNet on images of different rotations and transformations. Moreover, existing studies did not compare CapsNet and other CNN-based techniques in terms of their ability to recognize randomly transformed and rotated images. This is quite necessary, as one of the core advantages of CapsNet over CNN is its resistance to image rotations and transformations [2], as well as its ability to produce excellent results when trained on small datasets. This study aims to bridge some of the highlighted gaps. The main contributions of this study are highlighted in Section 1. 

**Table 1:** Summary of related studies.

Ref.	Method	Dataset	Performance
AbouEl-Magd et al. [4]	VGG16, CapsNet, SMOTE, and Gaussian optimization algorithm.	SIRM Dataset consisting of 219 COVID-19 chest X-ray images, 1341 standard images, and 1345 viral pneumonia images.	Classification accuracy of 96.58% and F1-score of 97.08%.
Saif et al. [6]	CapsNet, concatenation of parallel convolutional blocks of different filter sizes, concatenation of capsule layers.	POCUS dataset consisting of 64 videos [30]. A second dataset consisting of 1142 COVID-19 sample, 1332 normal images, and 1355 viral pneumonia images from different sources. A third dataset consisting of 230 COVID-19 images, 1064 normal images, and 1036 viral pneumonia images from different sources.	Classification accuracy of 98.3%, 99.0%, and 98.9%.
Toraman et al. [7]	Introduced a novel CapsNet architecture for COVID-19 diagnosis.	Dataset consist of 231 COVID-19 X-ray images [8], 1050 no findings images [9], and 1050 pneumonia images [9].	Classification accuracy of 84.22% and 97.24% for multi-class and binary class, respectively.
Tiwari et al. [10]	Proposed a hybrid technique for COVID-19 diagnosis. The framework consists of CapsNet and VGG16.	Dataset consist of 219, 1345, and 1341 radiography images of COVID-19, pneumonia, and normal conditions, respectively [11].	Classification accuracy of 97% for binary classification and 92% for multi-class classification.
Afshar et al. [12]	Designed a CapsNet framework. No data augmentation was used. Data imbalance technique was introduced.	Dataset consist of 358 CXR COVID-19 images, 8,066 8,066 normal, and 5538 non-COVID-19 images.	Accuracy, sensitivity, specificity, and AUC of 95.7%, 90%, 95.8%, and 0.97, respectively.
Heidarian et al. [13]	Designed a two-stage framework for COVID-19 diagnosis using CapsNet and volumetric CT images. Infected slices of CT images are detected in the first stage, and the images are classified in the second stage.	Dataset consist of 171, 60, and 76 COVID-19, CAP, and normal volumetric CT images, respectively.	Accuracy, sensitivity, specificity, and AUC of 90.82%, 94.55%, 86.04%, and 0.98, respectively.
Quan et al. [14]	Proposed a DL-based framework for COVID-19 diagnosis using DenseNet121 and CapsNet. The framework uses TernausNet for segmentation, DenseNet121 for feature extraction, and CapsNet for classification.	Dataset contains 781, 2917, 2884, and 2850 COVID-19, normal, pneumonia (virus), and pneumonia (bacteria) X-ray images.	Accuracy, sensitivity, and F1-score of 90.7%, 96%, and 90.9%, respectively.
Qi et al. [17]	Proposed a four-module pipeline for COVID-19 diagnosis. The first two modules are used for lung segmentation and selection of slices with lesions. The last two modules use CapsNet, ResNet50, and DenseNet121 for slice-level and patient-level prediction.	Dataset contains 161 CT images with COVID-19 and 100 CT images with CAP.	Classification accuracy and AUC of 97.1% and 0.992, respectively, for slice-level prediction. Classification accuracy of 100% for patient-level prediction.
Attallah [19]	Designed a novel method for building classification models for CT and X-ray images. The method uses four radiomic methods, three DL models, one feature reduction technique (FWHT), and one feature combination technique (FWHT).	Two datasets were used in the study. Dataset 1 consists of 1230 non-COVID-19 and 1252 COVID-19 CT scans. Dataset 2 consist of 1784 COVID-19 X-ray images, 1754 healthy X-ray scans, and 1345 X-Ray scans of people with pneumonia.	The technique was evaluated on a CT and X-ray dataset, and it produced 99.4% and 99% on the two datasets.
Mercaldo et al. [20]	VGG16.	Dataset consists of 18,000 CT images.	Classification accuracy of 95%.
Shah et al. [21]	DenseNet169, VGG16, ResNet50, InceptionV3, and VGG19.	Dataset consists of 738 CT images.	Classification accuracy of 94.52%.
Attallah and Samir [22]	ResNet50, ResNet101, ResNet18, DWT, and SVM.	Dataset 1 consist of 5152 normal 3D CT scans and 6012 COVID-19 images. Dataset 2 consist of 1252 COVID-19 and 1230 non-COVID-19 CT images.	The technique was evaluated on two datasets, and it achieved a classification accuracy of 99.33% and 99.7%, respectively.
Attallah [23]	ResNet50, ResNet101, ResNet18, discrete wavelet transform, and gray-level covariance matrix.	Dataset consist of 1252 COVID-19 and 1230 non-COVID-19 CT images.	Classification accuracy of 99.60%.
Zhao et al. [24]	Modified ResNet architecture	194,922 CT images.	Classification accuracy: 99.2%
Shankar and Perumal [25]	Gaussian filtering, local binary pattern model, InceptionV3, MLP classifier.	X-ray dataset consisting of 27 normal, 220 COVID-19, 11 SARS, and 15 Pneumocystis X-ray images.	Classification accuracy: 94.08%
Marios et al. [26]	Performance analysis of ResNet50, ResNet101, DenseNet121, DenseNet169, and InceptionV3.	11,956 COVID-19, 10,701 normal, and 11,263 pneumonia X-ray images.	ResNet101 achieved the best classification accuracy of 96%.

## 3. Methodology

**Figure 1:**
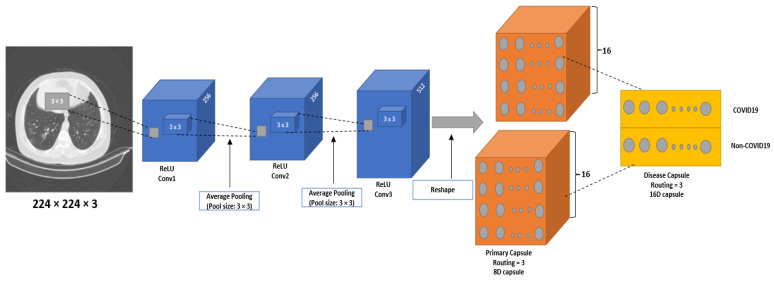
The proposed CapsNet architecture (CapsNetCovid).

This study proposes a CapsNet architecture for COVID-19 diagnosis (CapsNetCovid). The architecture is shown in Figure 1. The same model was used for the CT and X-ray images. The model consists of convolutional layer, primary capsule layer, and digit capsule layer. The convolutional layer is used to extract features from images, the primary capsule layer is used to learn different image parts and features of an image (such as orientation, size, pose, texture, etc.) and the spatial relationships between the parts. The digit capsule layer is used to perform the final classification. 

Specifically, the proposed model consists of three convolutional layers, sixteen primary capsule layers, and one digit capsule layer. Three convolutional layers were added to the network after performing experiments with different number of layers, kernels, and filter sizes. The convolutional layers help to extract effective and informed features for the primary capsule. The first and second convolutional layer consists of 256 kernels of size 3 × 3 with a stride of 1. The third convolutional layer consist of 512 kernels of size 3 × 3 with a stride of 2. The ReLU activation function is used for all the layers. The ReLU activation function is used to introduce non-linearity to the model and handle the vanishing gradient problem. 

Initially, images are passed through the three convolutional layers. The images are resized to 224 × 224 after experimenting with different image sizes. The output from the convolutional layer is passed to 16 primary capsule layers, where each capsule contains 8D vectors. The capsule layer applies convolutional operation with 9 × 9 kernel, and then squash the output to obtain a capsule. The output of the capsule layer is passed to a digit layer, containing 16D vectors per class. The layer is used to classify the CT images into two classes (COVID-19 and normal) and the X-ray images into three classes (COVID-19, normal, and pneumonia). 

Another CNN model was designed in this study for the purpose of comparison. The CNN model consists of two convolutional layers, one fully connected layer, and one output layer. The proposed architecture was also compared with DenseNet121 and ResNet50. The output of DenseNet121 and ResNet50 was passed through two fully connected layers, and one output layer. The output layer consists of two neurons for the binary classification, and three neurons for the multi-class classification. The pooling and dropout layer was also used to improve the computation speed and prevent overfitting. Note that only the added layers were finetuned. The number of layers and parameters for the CapsNet model, CNN model, and the two pre-trained models were selected after performing the series of experiments. More information about the parameters is presented in Table 2, Table 3 and Table 4.

Different experiments were performed to evaluate the efficacy of CapsNetCovid. Firstly, CapsNetCovid was trained on 80% of the dataset and evaluated on the remaining 20%. Twenty percent of the training set was reserved for validation. After training, the trained CapsNetCovid was saved and used in the subsequent experiments. During the other experiments, the saved CapsNet model was evaluated on the eight augmented datasets. Note that the augmented datasets were not used to train CapsNetCovid; they were only used to evaluate the pre-trained CapsNetCovid. We did this to assess CapsNetCovid’s ability to distinguish precisely between standard, flipped, shifted, and rotated images. Additionally, we wanted to evaluate the CapsNet’s ability to recognize augmented images, even if it was not exposed to such images during training. The same procedure was carried out for CNN, DenseNet121, and ResNet50. The models were trained, validated, and tested on the original datasets. After training, their trained weights were saved and evaluated on the eight augmented datasets. 

### 3.1. Dataset

Two types of datasets are used in this study. The first dataset type consists of standard images, while the second dataset type consists of augmented/transformed images. Standard images/datasets in this study refers to images/datasets that are not transformed (rotated or shifted).

#### 3.1.1. Standard Dataset

**Figure 2:**
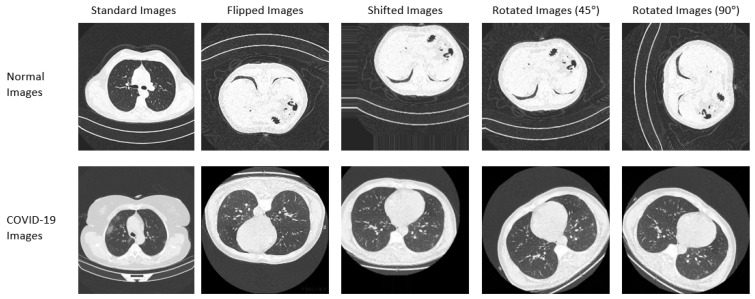
Samples of standard and augmented CT images used for training.

Two datasets with standard images were used in this study. The first dataset was obtained from different sources, including China National Center for Bio-information [31], National Institutes of Health Intramural Targeted Anti-COVID-19 [32], Negin Radiology Medical Center [33], Union Hospital and Liyuan Hospital of Huazhong University of Science and Technology [34], COVID-19 CT Lung and Infection Segmentation initiative [35], and the Radiopaedia collection [36]. The dataset (called COVID-Net CT-2) was created by Gunraj et al. [37]. Readers are referred to [37] for more information on the dataset. A subset of the COVID-Net CT-2 dataset is used in this study. Samples of the dataset are shown in Figure 2. The dataset consists of 14,000 CT images (9000 COVID-19 images and 5000 non-COVID-19 images). The second dataset was created by some researchers at the university of Qatar [38,39]. The dataset consists of 3616 COVID-19 X-ray images, 10,192 normal X-ray images, and 1345 pneumonia X-ray images. The dataset is publicly available and it can be downloaded from [40].

#### 3.1.2. Augmented Datasets 

**Figure 3:**
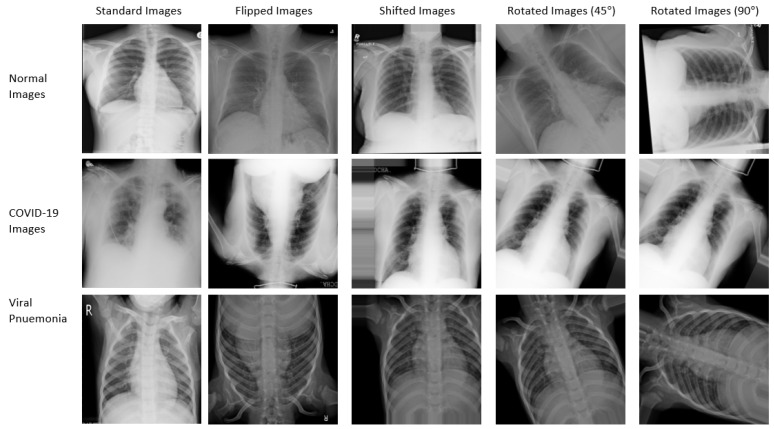
Samples of standard and augmented X-ray images used for training.

Eight new augmented datasets were generated from the original CT and X-ray datasets. The Keras ImageDataGenerator class was used to generate the augmented datasets. The first four augmented datasets consist of 14,000 randomly flipped CT images, 14,000 randomly shifted CT images, 14,000 CT images rotated randomly by 45 degrees, and 14,000 CT images rotated randomly by 90 degrees. The last four augmented datasets consist of 15,153 randomly flipped X-ray images, 15,153 randomly shifted X-ray images, 15,153 X-ray images rotated randomly by 45 degrees, and 15,153 X-ray images rotated randomly by 90 degrees. More details on the dataset are provided in Table 5. Additionally, the samples from the CT and X-ray standard and augmented dataset are shown in Figure 2 and Figure 3, respectively.

During the pre-processing stage, the images’ pixel values were converted to the range 0 to 1 by dividing them by 255. This value was used because 255 is the maximum possible pixel value for an image. The images were also resized to 224 *×* 224 and used as inputs to the CapsNet model. Eighty percent of the dataset was used for training, while the remaining twenty percent was used to test the model. During training, 20 percent of the training images was used to validate the training performance. All the experiments were conducted on a computer cluster. The cluster computer had the following specifications: 2 × Intel Xeon E5-2697A v4 processors with 512 GB of 2.4 GHz DDR4 memory. 

### 3.2. Performance Measures

Five performance measures 3454 used to evaluate the performance of the models, namely accuracy, precision, sensitivity, F1-score, and area under the ROC curve (AUC-ROC). The performance metrics can be calculated using Equations (1)–(4). The five metrics are influenced by the number of true negatives (*TN*s), true positives (*TP*s), false negatives (*FN*s), and false positives (*FN*s).

(1) $$\mathrm{Accuracy}=\frac{\left(TN+TP\right)}{\left(TN+TP+FN+FP\right)}\times 100$$

(2) $$\mathrm{Sensitivity}=\frac{TP}{TP+FN}\times 100$$

(3) $$\mathrm{Precision}=\frac{TP}{TP+FP}\times 100$$

(4) $$F1-\mathrm{Score}=\frac{2*Precision*Sensitivity}{Precision+Sesisitivity}$$

AUC-ROC is a measure showing the efficacy of a model in separating different classes. A high AUC indicates that the model is performing well, while a low AUC indicates otherwise.

## 4. Results and Discussion

Different experiments were performed to evaluate the performance of the proposed CapsNet model. This section presents the results and discussion. This section also presents a comparative analysis between CapsNetCovid and CNN, ResNet50, DenseNet121, and two existing studies.

### 4.1. Performance of CapsNetCovid for Binary Classification

**Figure 4:**
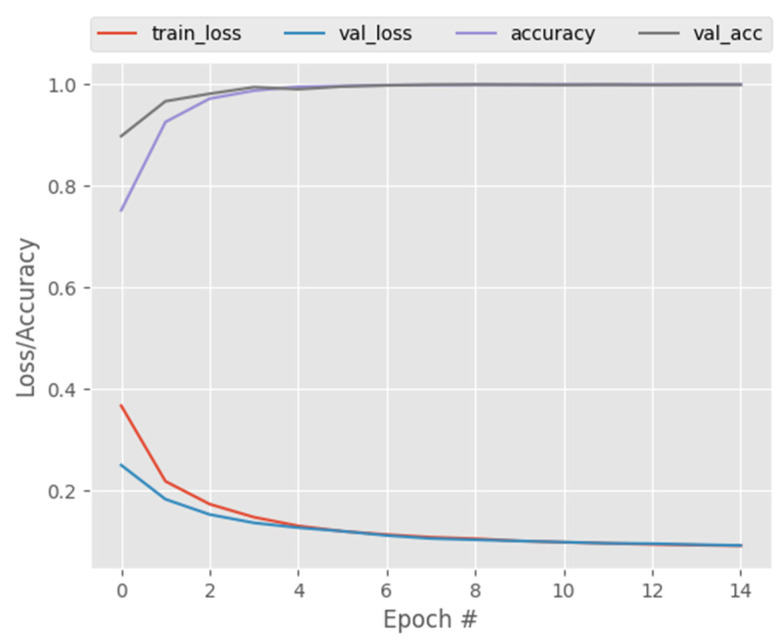
CapsNetCovid training and validation performance.

Table 6, Table 7, Table 8, Table 9 and Table 10 and Figure 4 show the performance of CapsNetCovid on COVID-19 CT scans. As shown, the CapsNet achieved a test accuracy of 99.929%. This shows that CapsNetCovid misclassified less than 0.1% of the CT images in the test dataset. Table 7 and Table 8 show the precision and sensitivity produced by the CapsNet during evaluation. The CapsNet achieved a precision, sensitivity and F1-score of 99.887%, 100%, and 99.316%, respectively. The sensitivity of 100% shows that the proposed model correctly classified all the COVID-19 samples, making them a good fit for medical diagnosis. It is crucial in the medical field to develop a model with a high degree of sensitivity. The precision of 99.887% shows the quality and completeness of the predictions. It confirms that all the COVID-19 samples were correctly predicted. The F1-score of 99.316% shows that the proposed CapsNet model correctly predicted 99.316% of the COVID-19 and normal samples across the evaluated dataset. This is quite admirable, as there is a good balance between the prediction of COVID-19 and normal samples in the dataset.

**Figure 5:**
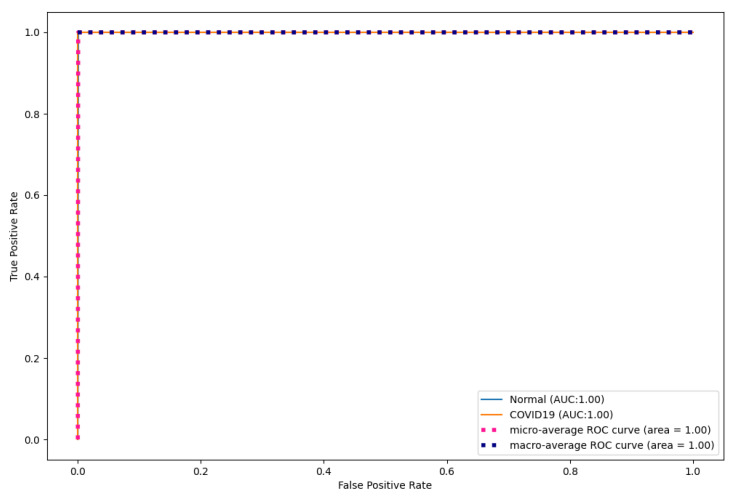
CapsNet ROC curves for CT images.

Table 10 shows the AUC scores produced by the proposed model. Furthermore, Figure 5 shows the AUC curves and their macro average with AUC scores. As shown, the proposed model performed well with an AUC of 100% for the two classes. This shows that the proposed model correctly distinguished all the COVID-19 and normal CT images in the original dataset. The proposed model is useful to medical practitioners because it correctly classifies all the COVID-19 and normal classes. A false positive result can lead to unnecessary procedures and treatments, while a false negative result can prevent a patient from receiving the necessary treatment, which can lead to the death of a patient.

### 4.2. Performance of CapsNetCovid on Augnemted Dataset for Binary Classification

Table 6, Table 7, Table 8, Table 9 and Table 10 also show the performance of CapsNetCovid on the augmented dataset. As shown, CapsNetCovid produced a classification accuracy of 71.075%, 84.935%, 87.114%, and 80.5844% for the RandomShift, RandomFlip, Rotated_45, and Rotated_90 datasets, respectively. The results show that the CapsNet is able to correctly identify a significant proportion of the augmented variants of the images it was trained on. The results also demonstrate the CapsNet’s resistance to image transformations and its ability to generate accurate results without additional data. Table 10 also shows that CapsNetCovid produced an AUC score of 0.61, 0.81, 0.81, and 0.72 for the RandomShift, RandomFlip, Rotated_45, and Rotated_90 datasets, respectively. This indicates that CapsNetCovid’s ability to reliably distinguish between COVID-19 and normal CT images decreased. The generalization performance of the CapsNet can be improved if it is exposed to augmented images during training. In addition, as demonstrated by the results, CapsNetCovid’s performance varies for various image transformations. The results also shows that the CapsNet is more robust at capturing randomly rotated and randomly flipped images than randomly shifted images. This shows that the robustness of the CapsNet depends on the type and degree of image transformation. More work is required to improve the generalization performance of CapsNet when applied to augmented medical images. This presents an opportunity for future research.

### 4.3. Comparative Analysis of CapsNetCovid with CNN-Based Techniques on Binary Classification

**Figure 6:**
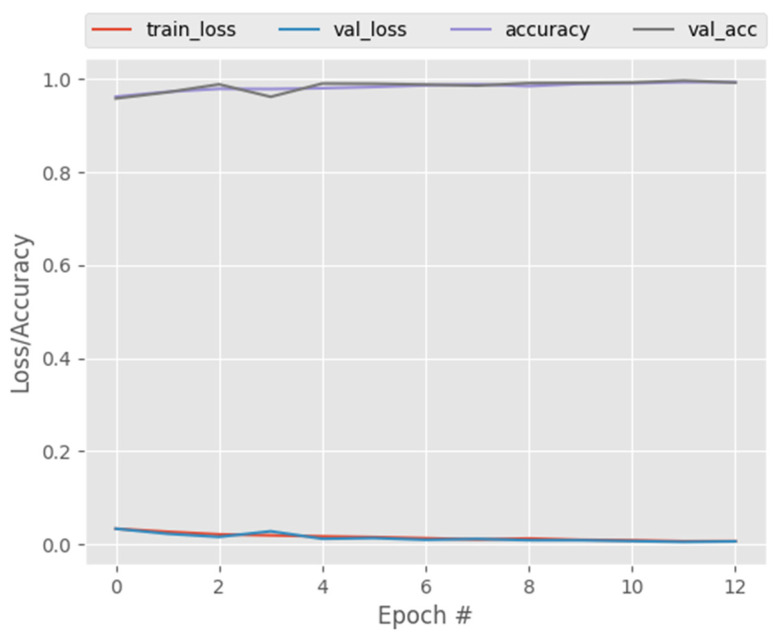
CNN training and validation performance.

**Figure 7:**
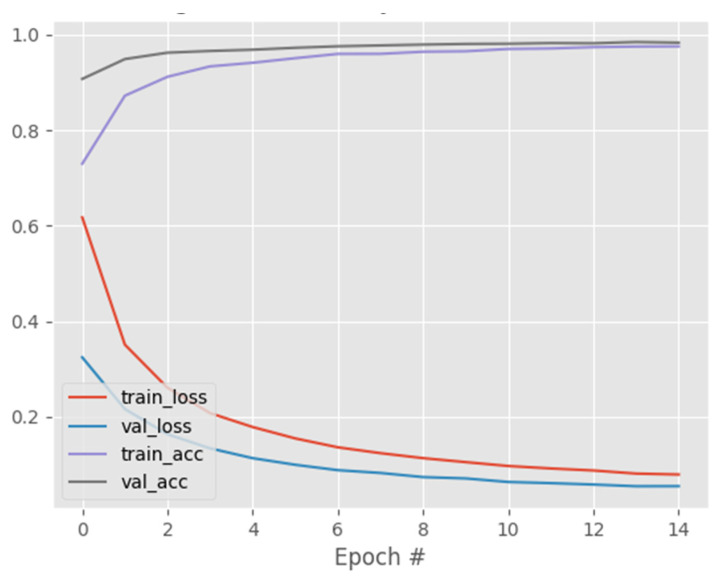
DenseNet121 training and validation performance.

**Figure 8:**
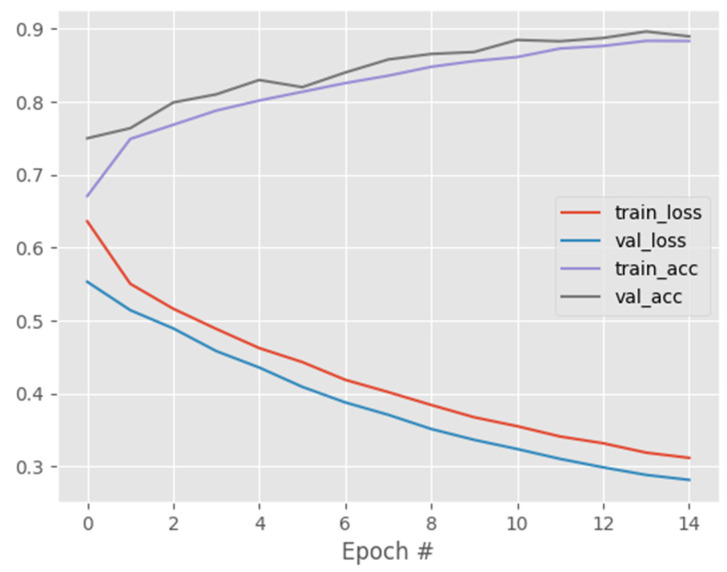
ResNet50 training and validation performance.

One of the key advantages of the CapsNet over CNN is its ability to capture affine rotations and transformations better than CNN. In view of this, we trained CNN, DenseNet121, and ResNet50 on the same COVID-19 dataset and compared their performance to that of CapsNetCovid. The results are shown in Table 6, Table 7, Table 8, Table 9 and Table 10 and Figure 6, Figure 7 and Figure 8. As shown in the table, CapsNetCovid outperformed CNN on the standard and rotated datasets. CapsNetCovid produced better classification accuracy, precision, sensitivity, and F1-score than CNN in most cases. This indicates that the CapsNet is more robust than CNN in identifying randomly rotated and transformed images without data augmentation. This is because the CNN model must be trained on all orientations of the images to achieve very good results. However, the CapsNet can detect and learn all orientations from a single image using a single capsule. In addition, it should be noted that the CapsNet is a recent DL algorithm. CNN existed before the CapsNet and has undergone numerous improvements over the years. Therefore, it is quite enthralling to see the CapsNet outperform CNN in most cases. 

CapsNetCovid was compared with two state-of-the-art CNN pre-trained models, namely DenseNet121 and ResNet50. The two models were finetuned on the COVID-19 datasets used in this study. After training, the finetuned models were saved and evaluated on the four augmented datasets. The results of the experiments are reported in Table 6, Table 7, Table 8, Table 9 and Table 10. As shown in the tables, CapsNetCovid produced better classification accuracy, sensitivity, precision, and F1-score than DenseNet121 and ResNet50 in the original dataset. CapsNetCovid also outperformed DenseNet121 and ResNet50 in the augmented datasets in most cases. In addition, the outcomes demonstrate that CapsNetCovid produced a higher AUC score than DenseNet121 and ResNet50 for the RandomFlip and Rotated_45 datasets. Additionally, it produced a higher AUC score than ResNet50 for the Rotated_90 dataset. This demonstrates that the CapsNet is superior to CNN at detecting transformations in images. Note that DenseNet121 and ResNet50 have already been trained on a large-scale dataset (ImageNet) containing over 1.2 million images. Nonetheless, the CapsNet still performed better than the two models. This demonstrates the capability of the CapsNet to handle small and augmented medical image datasets without data augmentation techniques. 

**Figure 9:**
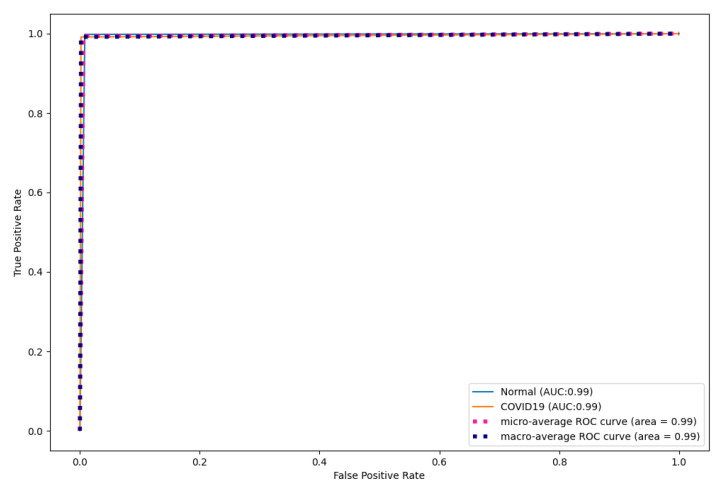
CNN ROC curves for CT images.

**Figure 10:**
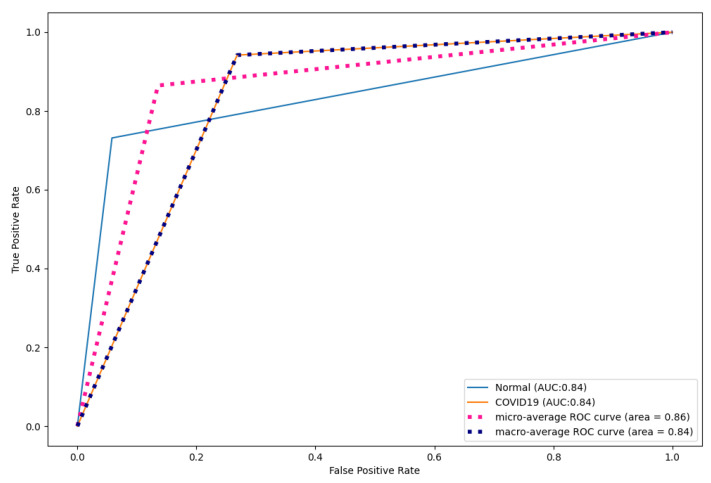
DesNet121 ROC curves for CT images.

**Figure 11:**
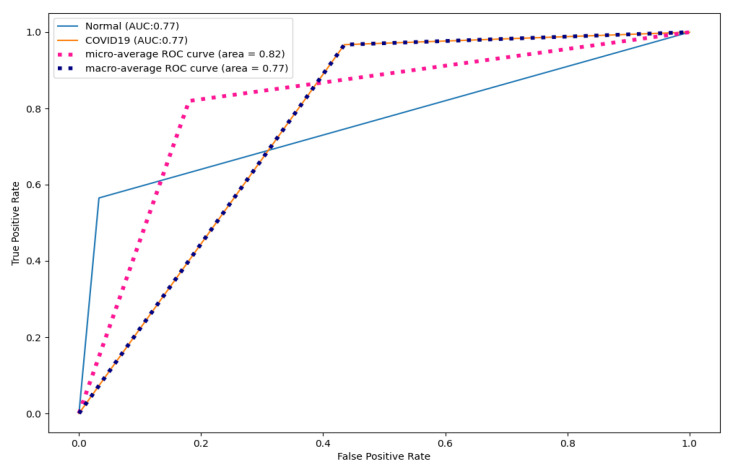
ResNet50 ROC curves for CT images.

Figure 9, Figure 10 and Figure 11 show the ROC curves for CNN, DenseNet121, and ResNet50. As shown, CapsNetCovid outperforms the AUC score of CNN, DenseNet121, and ResNet50 by 0.01%, 0.16%, and 0.23% for both normal and COVID-19 CT images. This shows that CapsNetCovid is more effective at distinguishing between positive and negative classes than the three compared CNN-based models.

### 4.4. Performance of CapsNetCovid on Multi-Class Classification

**Figure 12:**
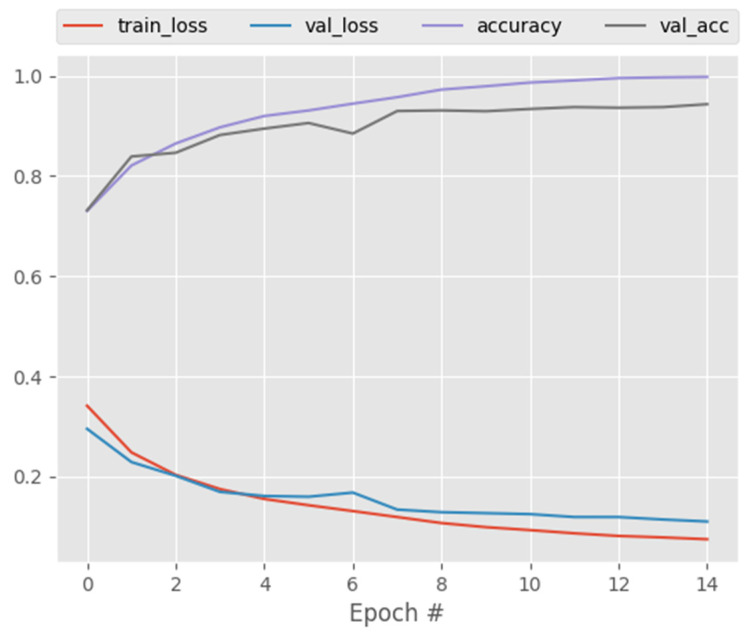
CapsNet training and validation performance for X-ray images.

**Figure 13:**
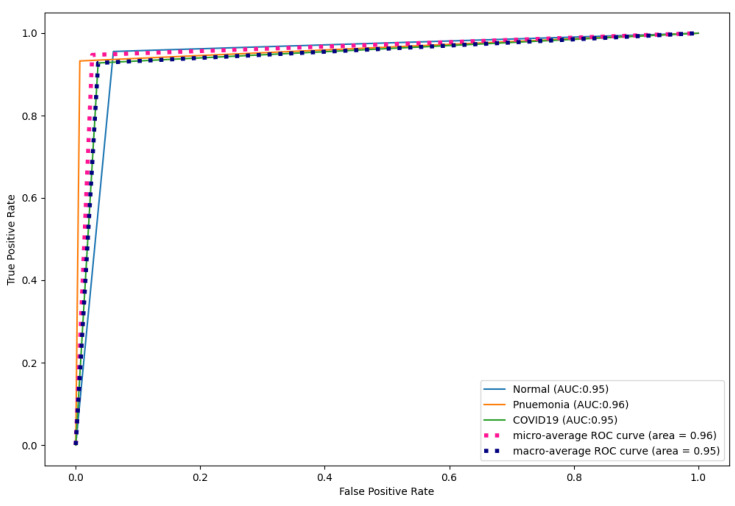
CapsNet ROC curves for X-ray images.

The proposed technique was applied to a dataset with three classes: COVID-19, normal, and pneumonia. Figure 12 and Table 11, Table 12, Table 13, Table 14 and Table 15 show the performance of CapsNetCovid on the multi-class dataset. As shown, CapsNetCovid achieved a classification accuracy, precision, sensitivity, and F1-score of 94.721, 93.864%, 92.947%, and 93.386%, respectively. The accuracy shows that the proposed model correctly predicted over 94% of the images in the dataset. Figure 13 shows that CapsNetCovid also produced an AUC score of 95.21%. This shows that the model has a strong ability in distinguishing between COVID-19, normal, and pneumonia X-ray images. CapsNetCovid correctly predicted 95% of normal X-ray scans, 96% of pneumonia scans, and 95% of COVID-19 X-ray scans. 

Table 12, Table 13 and Table 14 shows the precision, sensitivity, and F1-score of CapsNetCovid. As shown, CapsNetCovid produced a precision, sensitivity, and F1-score of 93.864%, 92.947%, and 93.386%, respectively. The high F1-score shows that the model has good generalization performance, and it performs well for normal, COVID-19 and pneumonia classes. The high sensitivity shows that the model correctly identified most of the COVID-19 and pneumonia classes. This is quite remarkable because it can be catastrophic to incorrectly diagnose a patient with COVID-19 or pneumonia. Medical practitioners prefer models with high sensitivity than models with high accuracy. The high precision shows that the CapsNetCovid model is 93.864% correct when it predicts an image to be COVID-19 or pneumonia.

It was observed that the performance of CapsNetCovid reduced from 99.929% to 94.721% when applied to multi-class classification. This decrease could be because of the quality of images in the dataset or the change in image modality. This may indicate that the CapsNet performs better on CT images compared to X-ray images. This reduction may also be because of the multi-class dataset. This may indicate that CapsNet performs better on binary classification compared to multi-class classification. More experiments are required to confirm the reason(s) for the decrease in performance. Overall, the proposed model performed well on the original X-ray images.

### 4.5. Comparative Analysis of CapsNetCovid with CNN-Based Techniques on Multi-Class Classification

**Figure 14:**
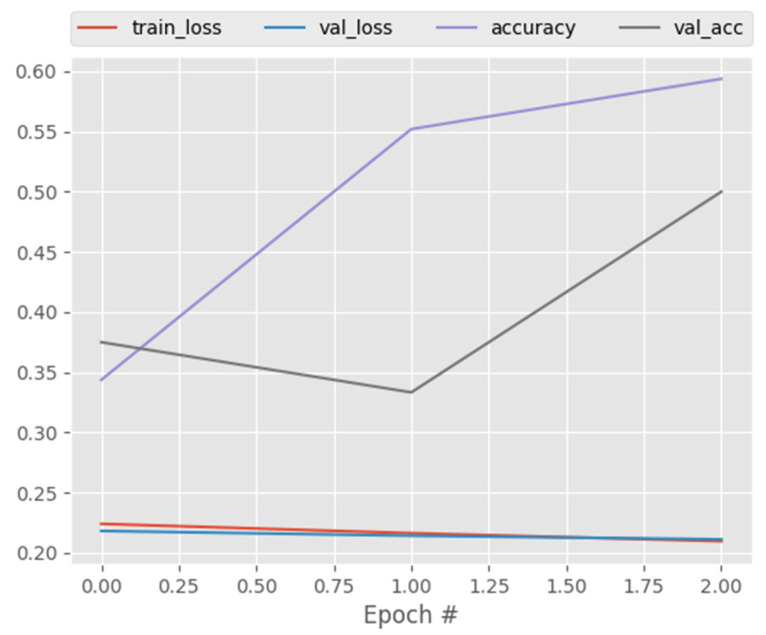
CNN training and validation performance for X-ray images.

**Figure 15:**
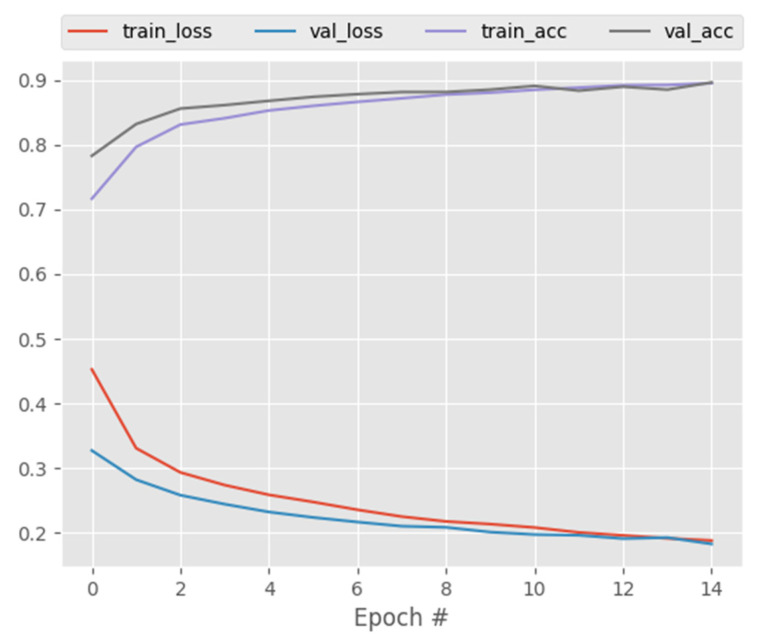
DenseNet121 training and validation performance for X-ray images.

**Figure 16:**
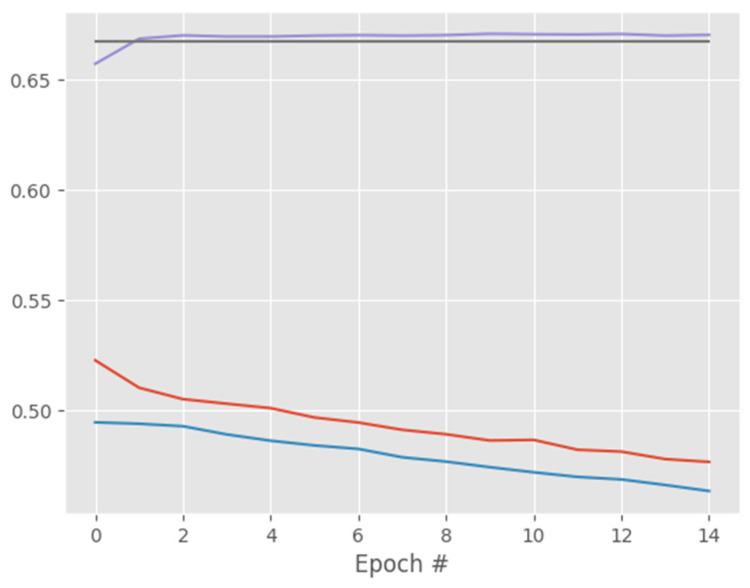
ResNet50 training and validation performance for X-ray images.

Figure 14, Figure 15 and Figure 16 and Table 11, Table 12, Table 13, Table 14 and Table 15 shows the performance of CNN, DenseNet121, and ResNet50 on the multi-class dataset. As shown, CapsNetCovid outperforms the three models in terms of classification accuracy and AUC score. It outperforms CNN, DenseNet121, and ResNet50 by 5.18%, 4.52%, and 26.36%, respectively. This shows that CapsNetCovid performs better than CNN in correctly distinguishing between COVID-19, pneumonia, and normal X-ray images without using data augmentation. It also shows that the proposed technique outperformed the compared CNN-based techniques in terms of correctly identifying COVID-19 and pneumonia cases. The proposed model will be a good fit for medical practitioners as its predictions for COVID-19, pneumonia, and normal X-ray images are satisfactory.

Note that DenseNet121 and ResNet50 are pre-trained on the ImageNet dataset containing over 1.2 million images. This shows that CapsNet does not need to be trained on large-scale datasets to outperform CNN-based models. The results also show that CapsNetCovid produced higher F1-score, precision, sensitivity, and AUC score than the compared CNN-based techniques in most cases. This indicates that the proposed technique has a better ability to correctly predict COVID-19 and pneumonia X-ray scans compared to CNN, DensNet121, and ResNet50. This shows that the CapsNet will be more acceptable to medical practitioners compared to CNN, especially when working with small datasets, which is mostly the case for medical image datasets.

**Figure 17:**
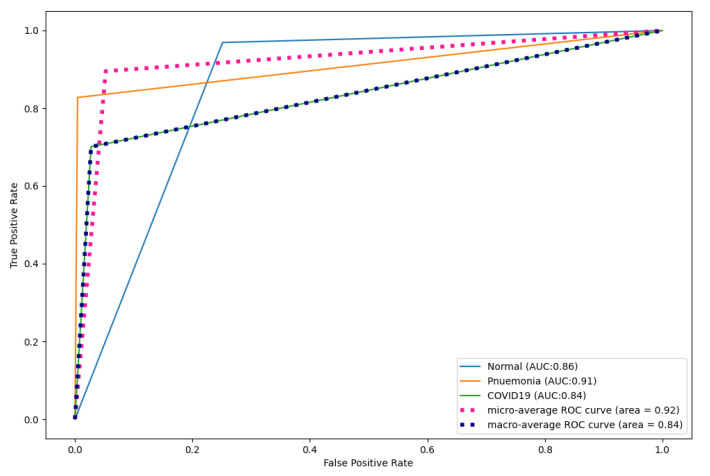
CNN ROC curves for X-ray images.

**Figure 18:**
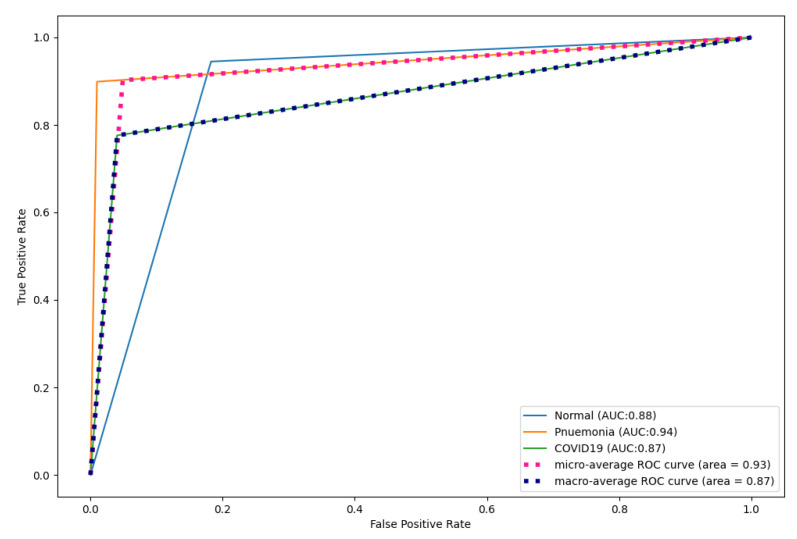
DesNet121 ROC curves for X-ray images.

**Figure 19:**
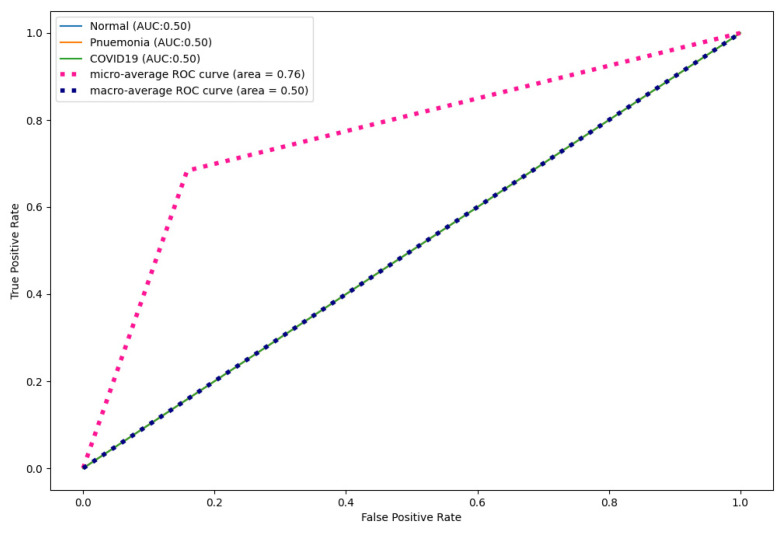
ResNet50 ROC curves for X-ray images.

Figure 17, Figure 18 and Figure 19 show the ROC curves produced by CNN, DenseNet121, and ResNet50 for multi-class classification. As shown, CapsNetCovid outperformed CNN by 0.09%, 0.05%, and 0.11% for normal, pneumonia, and COVID-19 images, respectively. CapsNetCovid outperformed DenseNet121 by 0.07%, 0.02%, and 0.08% for normal, pneumonia, and COVID-19 images, respectively. CapsNetCovid outperformed ResNet50 by 0.45%, 0.46%, and 0.45% for normal, pneumonia, and COVID-19 images, respectively. This shows that CapsNetCovid is more effective at correctly predicting COVID-19, pneumonia, and normal X-ray images than CNN, DenseNet121, and ResNet50.

### 4.6. Performance of CapsNetCovid on Augmented Dataset for Multi-Class Classification

As aforementioned, the proposed technique was evaluated on four augmented X-ray datasets containing 15,153 randomly flipped, randomly rotated, and randomly shifted X-ray images. The results are reported in Table 11, Table 12, Table 13, Table 14 and Table 15. As shown, the performance of CapsNetCovid decreased when evaluated on the augmented images. This is obviously because the model was not exposed to any of the augmented images during training. CapsNetCovid was anticipated to successfully recognize a larger percentage of the augmented version of the images it was trained on. However, as shown in the results, that was not the case. This shows that the robustness of CapsNet to affine transformations requires improvement, especially for multi-class classification. This is an opportunity for future research.

As shown in Table 11, Table 12, Table 13, Table 14 and Table 15, the performance of CapsNetCovid on randomly flipped, randomly rotated, and randomly shifted images varies. It achieved a higher classification accuracy for randomly flipped and randomly rotated images. This shows that the CapsNet is more resistant to randomly flipped and rotated images compared to randomly shifted images. CapsNetCovid also produced higher AUC score for randomly flipped and rotated images. This shows that it correctly predicted more randomly flipped COVID-19 and pneumonia images compared to normal images. The results also show that CapsNetCovid performed better on images that are randomly rotated by 45 degrees compared to images that are rotated by 90 degrees. This shows that the robustness of the CapsNet for image rotation is limited by the degree of image rotation.

### 4.7. Comparative Analysis of CapsNetCovid with CNN-Based Techniques on Multi-Class Classification

Table 11, Table 12, Table 13, Table 14 and Table 15 also show the performance of CNN, DenseNet121, and ResNet50 on augmented X-ray images. As shown in the results, the performance of the three models also decreased. CapsNetCovid produced better accuracy than CNN for randomly flipped and randomly rotated images. Furthermore, although DenseNet121 and ResNet50 produced higher classification accuracy than CapsNetCovid, the proposed model produced better precision, sensitivity, and F1-score than DenseNet121 and ResNet50. This shows that CapsNet is more robust than CNN-based techniques in correctly identifying COVID-19 and pneumonia images. The high classification accuracy of DenseNet121 and ResNet50 is most likely because the two models were pre-trained on over 1.2 million normal and augmented images. This suggests that data augmentation can be used to improve the robustness and generalization performance of CapsNet for image transformations. This can be confirmed from the performance of the CNN model. The CNN model was not previously trained on the augmented images, and it performed poorer than CapsNet, DenseNet121, and ResNet50. 

Furthermore, as shown in the results, CapsNetCovid outperform CNN, DenseNet121, and ResNet50 in terms of precision, sensitivity, and F1-score. This shows that CapsNet is more robust for image rotations and affine transformation than the compared CNN-based techniques. Figure 13 shows the ROC curves of CapsNetCovid for the three classes and their macro average. As shown, the proposed model produced a better AUC score for standard images compared to augmented images. This shows that the performance of the CapsNet can be improved if it is exposed to augmented images during training. The ROC curves for CNN, DenseNet121, and ResNet50 are shown in Figure 17, Figure 18 and Figure 19. As shown, CapsNetCovid produced a better AUC score than CNN and ResNet50. This shows that it outperforms the two models in correctly predicting COVID-19 and pneumonia images. 

### 4.8. Comparison of CapsNetCovid with Related Studies

The proposed technique is compared with existing state-of-the-art COVID-19 diagnosis techniques. The technique is compared with 10 binary classification techniques and 11 multi-class classification techniques. The results are reported in Table 16 and Table 17. As shown in the tables, the proposed technique outperformed all the compared techniques for binary classification and most of the techniques for multi-class classification. It is noteworthy to highlight that some of the compared techniques combined CNN pre-trained models with CapsNet. Notwithstanding, the proposed CapsNetCovid model still outperformed most of them. As an example, Tiwari and Anurag [41] proposed a CapsNet architecture for COVID-19 diagnosis from CT scans. They hybridized different CNN pre-trained models with a CapsNet. As shown in the results, CapsNetCovid performed slightly better than DenseCapsNet. It should be noted that DenseCapsNet is an aggregation of CapsNet and DensNet121, implying that it is already pre-trained on the ImageNet dataset with millions of images. Despite this, CapsNetCovid still produced comparable results to DenseCapsNet. Some studies combined CNN and SVM, CNN and CapsNet, optimization techniques and InceptionV3; nevertheless, the proposed model still outperformed them.

### 4.9. Summarized Results and Deductions 

Different experiments were performed in this study, and their results are presented in Section 4.1, Section 4.2, Section 4.3, Section 4.4, Section 4.5, Section 4.6, Section 4.7 and Section 4.8. As shown in the results, CapsNetCovid performed differently for both CT and X-ray images. The summary of the all the results is presented in this section. Deductions from the results are also presented in this section.

- The results show that CapsNetCovid performs well on standard X-ray and CT images. It produced better accuracy when trained and evaluated on CT images and binary classification. Its performance slightly decreased when trained and evaluated on X-ray images and multi-class classification. Overall, the proposed technique produced very good accuracy, sensitivity, F1-score, and AUC score when trained on standard images without data augmentation. The proposed technique also performs well on small medical image datasets. This is because the CNN model must be trained on all orientations of the images to achieve very good results. However, CapsNet can detect and learn all orientations from a single image using a single capsule.
- The results show that CapsNet is able to correctly identify a large proportion of the augmented variants of the images it was previously trained on, especially for binary classification. This demonstrates the CapsNet’s resistance to image transformations and its ability to achieve good results without data augmentation techniques.
- The performance of the CapsNet decreased when evaluated on the augmented variants of images it was previously trained on. This decrease was higher for X-ray images and multi-class classification. This is an indication that the CapsNet is more resistant to image rotations and transformations for binary classification than multi-class classification.
- The results show that CapsNetCovid outperforms CNN, DenseNet121, and ResNet50 when trained and evaluated on CT and X-ray images without data augmentation. This indicates that CapsNet is an excellent choice when working with small dataset and binary and multi-class classification.
- CapsNet outperforms CNN, DenseNet121, and ResNet50 when evaluated on an augmented CT image dataset with two classes (binary classification). It outperforms the CNN-based techniques in terms of classification accuracy, sensitivity, F1-score, and AUC score. Furthermore, although DenseNet121 and ResNet50 outperform the CapsNet in terms of classification accuracy, the CapsNet produced better precision, sensitivity, and F1-score than CNN, DenseNet121 and ResNet50 when evaluated on an augmented X-ray dataset with three classes (multi-class classification). This shows that medical practitioners will favor the CapsNet over CNN due to the significance of high sensitivity and F1-score in the medical domain. The higher classification accuracy of DenseNet121 and ResNet50 is most likely because the two models are pre-trained on a dataset with over 1.2 million normal and augmented images. This suggests that data augmentation can be used to improve the performance of the CapsNet for multi-class classification.
- The results show that the CapsNet produces a better AUC score than CNN, DenseNet121, and ResNet50 for both binary and multi-class classification problems. This shows that the CapsNet has a better ability to distinguish between positive and negative classes, which is remarkable.

Overall, as shown in all the reported results, the proposed CapsNet model produced very good results for a small medical image dataset and it outperformed CNN, DenseNet121, and ResNet50 at classifying both standard and augmented CT and X-ray images. Moreover, Figure 4 and Figure 12 show the training and validation loss of CapsNetCovid. As shown, the training and validation loss and accuracy curves are nearly overlapping, indicating that there is no significant variance between the training and validation loss and accuracy. This shows that the CapsNet model did not overfit.

## 5. Conclusions

The COVID-19 pandemic remains a threat, with multiple waves causing significant damage to the health of millions of people around the world. This study developed a CapsNet model (named CapsNetCovid) for COVID-19 diagnosis using CT and X-ray images. The model achieved a classification accuracy, precision, sensitivity and F1-score of 99.929%, 99.887%, 100%, and 99.319%, respectively, for CT images. Moreover, it achieved a classification accuracy, precision, sensitivity, and F1-score of 94.721%, 93.864%, 92.947%, and 93.386%, respectively, for the X-ray dataset. CapsNetCovid was compared with a CNN model designed for the purpose of comparison, and it outperformed the model on both standard and augmented CT and X-ray images. CapsNetCovid was also compared with two state-of-the-art pre-trained models, namely DenseNet121 and ResNet50. CapsNetCovid outperformed the two models for the standard CT and X-ray image dataset. 

Moreover, the results show that CapsNetCovid is more resistant to image rotations and affine transformations than CNN, DenseNet121 and ResNet50 for CT and X-Ray images. Furthermore, the results show that the CapsNet is more resistant to image rotations and transformations for binary classification than multi-class classification. Furthermore, the results show that the CapsNet performs better when applied to randomly rotated and flipped images compared to shifted images. The results also suggest that data augmentation can be used to improve the performance of the CapsNet for multi-class classification. Data augmentation can also be used to improve the overall generalization performance of the CapsNet. Future research can focus on improving the generalization performance of the CapsNet and the robustness of the CapsNet for image rotations and transformations, especially for multi-class classification problems.

## Figures and Tables

**Table 2:** Training Parameters for CapsNetCovid.

N_Class	Epochs	LR	BS	N_Routing	Optimizer
2	15	0.001	64	3	Adam

**Table 3:** Parameters used for DenseNet121 and ResNet50.

Algorithm	Dropout Rate	Learning Rate	Pooling Layer	FC	BS	Optimizer	Epochs	Activation
DenseNet121	0.5	0.0001	Pool size = 4	Units = 96	64	Adam	15	ReLU
ResNet50	0.5	0.0001	Pool size = 5	Units = 80	64	Adam	15	ReLU

**Table 4:** CNN model parameters.

Layers	Values
Conv1	Filters = 64, kernel size = 5 × 5
Average Pooling layer	Pool size = 5 × 5
Conv2	Filters = 64, kernel size = 5 × 5
Average Pooling layer	Pool size = 3 × 3
Fully Connected layer 1	Units = 128
Drop out layer	Dropout rate = 0.5
Optimizer	Adam
Activation	ReLU
Learning rate	0.0001

**Table 5:** Dataset summary.

Dataset	Number of Images	Description
Standard CT Image Dataset	14,000	Images in this dataset are not augmented.
RandomlyFlip CT Image dataset	14,000	Images in this dataset are randomly flipped horizontally and vertically.
RandomShift CT Image dataset	14,000	Images in this dataset are shifted by a number of pixels to the left, right, and vertically. The width and height shift range are set to 0.2.
RandomRotated CT Image dataset (45 degree)	14,000	Images in this dataset are randomly rotated by 45 degrees.
RandomRotated CT Image dataset (45 degree)	14,000	Images in this dataset are randomly rotated by 90 degrees.
Standard X-ray Image Dataset	15,153	Images in this dataset are not augmented.
RandomlyFlip X-ray Image Dataset	15,153	Images in this dataset are randomly flipped horizontally and vertically.
RandomShift X-ray Image Dataset	15,153	Images in this dataset are shifted by a number of pixels to the left, right, and vertically. The width and height shift range are set to 0.2.
RandomRotated X-ray Image dataset (45 degree)	15,153	Images in this dataset are randomly rotated by 45 degrees.
RandomRotated X-ray Image dataset (45 degree)	15,153	Images in this dataset are randomly rotated by 90 degrees.

**Table 6:** Classification accuracy of CapsNetCovid, CNN, DenseNet121, and ResNet50.

Technique	Original Dataset (%)	RandomShift Dataset (%)	RandomFlip Dataset (%)	Rotated_45 Dataset (%)	Rotated_90 Dataset (%)
CapsNetCovid (Ours)	99.929	71.157	84.892	87.121	80.588
CNN	99.143	73.45	86.757	74.492	74.492
DenseNet121	97.750	81.521	82.136	81.885	79.342
ResNet50	88.2857	72.22	73.00	76.771	65.80

Key: RandomShift, randomly shifted images; RandomFlip, randomly flipped images; Rotated_45, randomly rotated images (45 degrees); Rotated_90, randomly rotated images (90 degrees).

**Table 7:** Precision of CapsNetCovid, CNN, DenseNet121, and ResNet50.

Technique	Original Dataset (%)	RandomShift Dataset (%)	RandomFlip Dataset (%)	Rotated_45 Dataset (%)	Rotated_90 Dataset (%)
CapsNetCovid (Ours)	99.887	96.056	87.556	98.511	95.433
CNN	98.815	82.163	73.066	69.934	64.147
DenseNet121	97.404	74.025	78.575	77.409	80.767
ResNet50	91.371	80.992	88.091	77.630	60.200

**Table 8:** Sensitivity of CapsNetCovid, CNN, DenseNet121, and ResNet50.

Technique	Original Dataset (%)	RandomShift Dataset (%)	RandomFlip Dataset (%)	Rotated_45 Dataset (%)	Rotated_90 Dataset (%)
CapsNetCovid (Ours)	100	70.125	88.789	84.158	78.820
CNN	99.829	64.367	64.840	64.047	64.433
DenseNet121	99.025	64.844	78.575	64.812	64.094
ResNet50	88.306	63.784	64.428	64.698	63.519

**Table 9:** F1-Score of CapsNetCovid, CNN, DenseNet121, and ResNet50.

Technique	Original Dataset (%)	RandomShift Dataset (%)	RandomFlip Dataset (%)	Rotated_45 Dataset (%)	Rotated_90 Dataset (%)
CapsNetCovid (Ours)	99.319	81.067	88.168	90.770	86.335
CNN	98.884	72.184	68.707	66.861	64.290
DenseNet121	98.208	69.131	70.990	70.553	71.471
ResNet50	91.937	71.365	74.724	70.576	61.815

**Table 10:** ROC score for CT Images.

Technique	Original Dataset (%)	RandomShift Dataset (%)	RandomFlip Dataset (%)	Rotated_45 Dataset (%)	Rotated_90 Dataset (%)
CapsNetCovid (Ours)	0.999	0.614	0.813	0.809	0.723
CNN	0.994	0.657	0.828	0.725	0.725
DenseNet121	0.836	0.770	0.761	0.761	0.727
ResNet50	0.766	0.644	0.631	0.706	0.635

**Table 11:** Classification accuracy for CapsNetCovid, CNN, DenseNet121, and ResNet50 for multi-class classification.

Technique	Original Dataset (%)	RandomShift Dataset (%)	RandomFlip Dataset (%)	Rotated_45 Dataset (%)	Rotated_90 Dataset (%)
CapsNetCovid (Ours)	94.721	51.393	60.7186	51.686	40.53
CNN	89.5414	54.332	55.892	50.146	0.4126
DenseNet121	90.234	84.162	82.1890	77.882	74.790
ResNet50	68.360	67.271	67.264	67.344	67.277

**Table 12:** Precision for CapsNetCovid, CNN, DenseNet121, and ResNet50 for multi-class classification.

Technique	Original Dataset (%)	RandomShift Dataset (%)	RandomFlip Dataset (%)	Rotated_45 Dataset (%)	Rotated_90 Dataset (%)
CapsNetCovid (Ours)	93.864	59.475	73.094	62.992	48.482
CNN	83.256	32.549	32.886	33.677	33.230
DenseNet121	87.329	33.111	34.517	34.319	34.171
ResNet50	33.333	33.333	33.333	33.333	33.333

**Table 13:** Sensitivity for CapsNetCovid, CNN, DenseNet121, and ResNet50 for multi-class classification.

Technique	Original Dataset (%)	RandomShift Dataset (%)	RandomFlip Dataset (%)	Rotated_45 Dataset (%)	Rotated_90 Dataset (%)
CapsNetCovid (Ours)	92.947	60.962	62.499	68.987	64.214
CNN	90.825	32.772	33.117	33.407	34.256
DenseNet121	88.929	33.126	34.645	34.133	34.159
ResNet50	22.787	22.468	22.222	22.222	22.222

**Table 14:** F1-Score for CapsNetCovid, CNN, DenseNet121, and ResNet50 for multi-class classification.

Technique	Original Dataset (%)	RandomShift Dataset (%)	RandomFlip Dataset (%)	Rotated_45 Dataset (%)	Rotated_90 Dataset (%)
CapsNetCovid (Ours)	93.386	52.698	61.641	56.059	40.708
CNN	86.501	29.090	29.609	28.206	25.190
DenseNet121	88.065	33.097	34.550	33.628	33.781
ResNet50	27.069	26.843	26.669	26.685	26.661

**Table 15:** AUC-ROC of CapsNetCovid, CNN, DenseNet121, and ResNet50 for multi-class classification.

Technique	Original Dataset (%)	RandomShift Dataset (%)	RandomFlip Dataset (%)	Rotated_45 Dataset (%)	Rotated_90 Dataset (%)
CapsNetCovid (Ours)	0.9521	0.6826	0.775	0.705	0.6059
CNN	0.869	0.6833	0.725	0.656	0.5822
DenseNet121	0.8971	0.8558	0.8274	0.838	0.7821
ResNet50	0.500	0.500	0.500	0.500	0.500

**Table 16:** CapsNetCovid versus existing COVID-19 diagnosis techniques for binary classification.

Technique	Model	Dataset	Accuracy (%)
Tiwari and Anurag [41]	DesneNet121 + CapsNet	1252 COVID-19 and 1230 non-COVID-19 CT images	99%
Prottoy et al. [42]	CNN + ML classifier ensemble	4600 COVID-19, 2300 non-COVID-19	98.91
Apostolopoulos et al. [43]	VGG19	224 COVID-19, 504 non-COVID-19	98.75
Narin et al. [44]	ResNet50	50 COVID-19, 50 non-COVID-19	98.0
Sethy et al. [45]	CNN + SVM	25 COVID-19, 25 non-COVID-19	95.3
Alqudah et al. [46]	CNN	1525 COVID-19, 3050 non-COVID-19	95.2
Dimeglio et al. [47]	DenseNet121	15,000 COVID-19 and non-COVID-19	99
Chakraborty et al. [48]	CNN	3797 COVID-19 and non-COVID-19	97.11
Toraman et al. [7]	CNN + CapsNet	1050 COVID-19, 1050 non-COVID-19	97.24
Sharma et al. [49]	CNN + CapsNet	3616 COVID-19, 11,537 non-COVID-19	97.69
**CapsNetCovid (Ours)**	**CapsNet**	**9000 COVID-19 and 5000 non-COVID-19**	**99.929**

**Table 17:** CapsNetCovid versus existing COVID-19 diagnosis technique for multi-class classification.

Technique	Model	Dataset	Accuracy (%)
Apostolopoulos et al. [43]	VGG19	224 COVID-19, 504 non-COVID-19, 700 pneumonia	93.48
Shain et al. [50]	CapsNet	55 COVID-19, 25 pneumonia, 78 CT images.	89.8
Afshar et al. [12]	CNN + CapsNet	COVID-19, bacterial, normal, non-COVID-19 viral	95.7
Mohammad et al. [51]	CNN	219 COVID-19, 51341 normal, 1345 pneumonia	96.69
Rahimzadeh et al. [52]	Xception, ResNet V2	180 COVID-19	80
Kim et al. [53]	ResNet, AlexNet, GoogleNet	69 COVID-19	80
Wang et al. [54]	CNN	53 COVID-19, 8066 healthy, 5526 pneumonia	93.3
Sharma et al. [49]	CNN + CapsNet	3616 COVID-19, 10,192 normal, 1345 pneumonia	96.47
Toraman et al. [7]	CNN + CapsNet	1050 COVID-19, 1050 normal, 1050 pneumonia	84.2
Shankar and Perumal [25]	Gaussian filtering, local binary pattern model, InceptionV3, MLP classifier	27 normal, 220 COVID-19, 11 SARS, and 15 pneumocystis images	94.08
Quan et al. [14]	DenseNet121 + CapsNet	781 COVID-19, 2917 normal, 2884 pneumonia, and 2850 pneumonia.	90.7
**CapsNetCovid (Ours)**	**CapsNet**	**3616 COVID-19, 10,192 Normal, 1345 pneumonia**	**94.721**

## Data Availability

Publicly available datasets were analyzed in this study. This data can be found here: [37,38,39].
